# The primate malaria parasites *Plasmodium malariae*, *Plasmodium brasilianum* and *Plasmodium ovale* spp.: genomic insights into distribution, dispersal and host transitions

**DOI:** 10.1186/s12936-022-04151-4

**Published:** 2022-05-03

**Authors:** Hans-Peter Fuehrer, Susana Campino, Colin J. Sutherland

**Affiliations:** 1 Institute of Parasitology, Department of Pathobiology, University of Veterinary Medicine Vienna, Veterinaerplatz 1, 1210 Vienna, Austria; 2 Department of Infection Biology, Faculty of Infectious & Tropical Diseases, London School of Hygiene & Tropical Medicine, London, UK

**Keywords:** *Plasmodium malariae*, *Plasmodium brasilianum*, *Plasmodium ovale curtisi*, *Plasmodium ovale wallikeri*, Host transitions

## Abstract

During the twentieth century, there was an explosion in understanding of the malaria parasites infecting humans and wild primates. This was built on three main data sources: from detailed descriptive morphology, from observational histories of induced infections in captive primates, syphilis patients, prison inmates and volunteers, and from clinical and epidemiological studies in the field. All three were wholly dependent on parasitological information from blood-film microscopy, and *The Primate Malarias”* by Coatney and colleagues (1971) provides an overview of this knowledge available at that time. Here, 50 years on, a perspective from the third decade of the twenty-first century is presented on two pairs of primate malaria parasite species. Included is a near-exhaustive summary of the recent and current geographical distribution for each of these four species, and of the underlying molecular and genomic evidence for each. The important role of host transitions in the radiation of *Plasmodium* spp. is discussed, as are any implications for the desired elimination of all malaria species in human populations. Two important questions are posed, requiring further work on these often ignored taxa. Is *Plasmodium brasilianum,* circulating among wild simian hosts in the Americas, a distinct species from *Plasmodium malariae*? Can new insights into the genomic differences between *Plasmodium ovale curtisi* and *Plasmodium ovale wallikeri* be linked to any important differences in parasite morphology, cell biology or clinical and epidemiological features?

## Background

In *The Primate Malarias* (1971), by Coatney et al*.* [1], detailed species comparisons are presented based on descriptive morphology of both blood and mosquito stages, the geographic distribution of each parasite and certain features readily measurable in induced human infections, including the estimated duration of the liver-stage, time to symptoms and fever periodicity. Much of this work was performed in prison inmates in Georgia, USA. In this paper, fifty years since, the focus on the geographic, genomic and genetic characteristics of four primate malaria species—one currently regarded as zoonotic in South American monkeys, *Plasmodium brasilianum*, and three malaria parasites of *Homo sapiens*, namely *Plasmodium malariae, Plasmodium ovale curtisi* and *Plasmodium ovale wallikeri*. An exhaustive bibliography of reported identification of these species since 1890, across the globe and in different primate hosts, will also be presented.

Over the last two decades, the analytical techniques of evolutionary biology and the task of reconstructing phylogenetic relationships within the genus have benefited greatly from the explosion in genomic data available for malaria parasites, and the now well-established practise of non-invasive faecal sampling of parasite genomic material from the faeces of wild primates [2]. This wealth of data provides new understanding of diversity both within and among the primate-infecting *Plasmodium* species, and points to the importance of transitions into new primate hosts. These transitions are gateways to the radiation of parasite species, but also act as genetic bottlenecks, as evidenced by reduced diversity among parasites in the new host [2, 3].

Among the homophilic species considered of clinical importance, a range of life history and transmission strategies are evident, and each of these strategies have their equivalent counterparts among the parasites of living simian hosts, and those of *Pan* and *Gorilla*. Thus, the majority of evolution leading to these diverse life histories occurred in the parasite lineages of non-human primates in the evolutionary past. However, as with *Plasmodium knowlesi,* the zoonotic potential of *P. brasilianum* shows that host transition can be a dynamic process operating over an extended time period, rather than a singular event, and understanding this in the present is essential to maintain effective malaria elimination strategies world-wide.

## *Plasmodium brasilianum*

### History & discovery

The first report of *P. brasilianum* is based on a finding in the blood of a bald uakari (*Cacajao calvus*) imported from the Brazil Amazonas region to Hamburg, Germany in 1908 [4]. Initial studies reported that *P. brasilianum* closely resembles *P. malariae,* and to be a relatively common parasite of New World monkeys in Panama and Brazil (reviewed in [1]).

### Distribution and known non-human primate hosts

Historically, natural infections of *P. brasilianum* were reported in various primates in Central and Southern America—Panama, Colombia, Venezuela, Peru, and Brazil. The spectrum of primate hosts (incl. sequence confirmed reports) is given in Table 1 [5–12], indicating that *P. brasilianum* has promiscuous host-specificity compared to other malaria parasites. Moreover, natural infections in humans have been reported from Venezuela [13].

**Table 1:** Non-human primate host spectrum of *Plasmodium brasilianum* (modified after Coatney 1971)

Host	Host Distribution	GenBank ID	References
Black howler (*Alouatta caraya*)	Argentina, Bolivia, Brazil, Paraguay		[5]
Brown howler (*Alouatta guariba*; Syn.: *A. fusca*)	Atlantic Forest—Brazil, Argentinia		[1]
Northern brown howler (*Alouatta guariba guariba*)	Brazil		[5]
Southern brown howler (*Alouatta guariba clamitans*)	Brazil, Argentinia	MF573323	[6]
Mantled howler (*Alouatta palliata*)	Colombia, Costa Rica, Ecuador, Guatemala, Honduras, Mexico, Nicaragua, Panama, Peru	KU999995	[1]
Red howler (*Alouatta seniculus*)	Venezuela, Colombia, Ecuador, Peru, Brazil, French Guyana	AF138878	[7]
Guatemalan black howler (*Alouatta pigra;* Syn.: *Alouatta villosa*)	Belize, Guatemala, Mexico		[1]
Gray-handed night monkey (*Aotus griseimembra*)	Colombia, Venezuela		[8]
Black-headed night monkey (*Aotus nigriceps*)	Brazil, Bolivia and Peru	KC906732	[9]
White-bellied spider monkey (*Ateles belzebuth*)	Colombia, Ecuador, Venezuela, Peru, Brazil		[5]
Peruvian spider monkey (*Ateles chamek*)	Peru, Brazil, Bolivia	KC906714	[9]
Black-headed spider monkey (*Ateles fusciceps*)	Colombia, Ecuador, Panama		[1]
Geoffroy's spider monkey (*Ateles geoffroyi*)	Central America incl. parts of Mexico, Colombia		[1]
Nicaraguan spider monkey (*Ateles geoffroyi geoffroyi*)	Nicaragua, Costa Rica		[1]
Hooded spider monkey (*Ateles geoffroyi grisescens*)	Panama, Colombia		[1]
Brown spider monkey (*Ateles hybridus*)	Colombia, Venezuela		[8]
Red-faced spider monkey (*Ateles paniscus*)	northern Brazil, Suriname, Guyana, French Guiana and Venezuela		[5]
Southern muriqui (*Brachyteles arachnoides*)	Brazilian states Paraná, São Paulo, Rio de Janeiro, Espírito Santo, Minas Gerais		[5]
Bald uakari (*Cacajao calvus*)	Brazil, Peru		[5]
Red bald-headed uakari (*Cacajao calvus rubicundus*)	Brazil		[5]
Masked titi (*Callicebus personatus*)	Brazil		[5]
White-headed marmoset (*Callithrix geoffroyi*)	Brazil		[10]
Collared titi (*Cheracebus torquatus;* Syn.: *Callicebus torquatus*)	Brazil (Amazonas)		[5]
White-fronted capuchin (*Cebus albifrons*)	Bolivia, Brazil, Colombia, Venezuela, Ecuador, Peru, Trinidad and Tobago		[1]
Colombian white-faced capuchin (*Cebus capucinus*)	Colombia, Ecuador		[1]
Panamanian white-faced capuchin (*Cebus imitator*)	Honduras, Nicaragua, Costa Rica, Guatemala, Belize, Panama		[1]
Varied white-fronted capuchin (*Cebus versicolor*)	Colombia		[8]
White-nosed saki (*Chiropotes albinasus*)	Brazil, Bolivia		[5]
Red-backed bearded saki (*Chiropotes chiropotes*)	North of the Amazon River and East of the Branco River, in Brazil, Venezuela and the Guianas	KC906730	[9]
Black bearded saki (*Chiropotes satanas*)	Brazil		[5]
Gray woolly monkey (*Lagothrix cana*)	Bolivia, Brazil, Peru	KC906726	[9]
Brown woolly monkey (*Lagothrix lagotricha*)	Colombia, Ecuador, Peru, Brazil		[5]
Brown-mantled tamarin (*Leontocebus fuscicollis*, Syn.: *Saguinus fuscicollis*)	Bolivia, Brazil, Peru		[11]
Golden-headed lion tamarin (*Leontopithecus chrysomelas*)	Brazil		[10]
Golden lion tamarin (*Leontopithecus rosalia*)	Brazil		[10]
Santarem marmoset (*Mico humeralifer*)	Brazil		[10]
Gray's bald-faced saki (*Pithecia irrorata*)	Colombia, Bolivia, Peru, Brazil	KC906717	[9]
Monk saki (*Pithecia monachus*)	Brazil, Peru, Ecuador Colombia		[5]
White-faced saki (*Pithecia pithecia*)	Brazil, French Guiana, Guyana, Suriname, Venezuela		[5]
Brown titi (*Plecturocebus brunneus*; Syn.: *Callicebus brunneus*)	Brazil, Peru, and Bolivia		[9]
Chestnut-bellied titi (*Plecturocebus caligatus*, Syn.: *Callicebus caligatus*)	Brazil	JX045640	[12]
Red-bellied titi (*Plecturocebus moloch*)	Brazil	KC906723	[9]
Hershkovitz's titi (*Plecturocebus dubius;* Syn.: *Callicebus dubius*)	Bolivia, Brazil, Peru	JX045642	[12]
Emperor tamarin *(Saguinus imperator*)	Bolivia, Brazil, Peru	KY709306	[11]
Golden-handed tamarin (*Saguinus midas*)	Brazil, Guyana, French Guiana, Suriname		[5]
Geoffroy's tamarin (*Saguinus geoffroyi*)	Panama, Colombia		[11]
Martins's tamarin (*Saguinus martinsi;* both subspecies: *Saguinus martinsi martinsi, Saguinus martinsi ochraceous*)	Brazil		[10]
Black tamarin (*Saguinus niger*)	Brazil		[11]
Tufted capuchin (*Sapajus apella*)	Brazil, Venezuela, Guyanas, Colombia, Ecuador, Bolivia, Peru	KC906715	[9]
Blond capuchin (*Sapajus flavius*)	Brazil	KX618476	**
Large-headed capuchin (*Sapajus macrocephalus*; Syn.: *Sapajus apella macrocephalus*)	Bolivia, Brazil, Colombia, Ecuador, Peru		[5]
Robust tufted capuchin (*Sapajus robustus*)	Brazil		[5]
Golden-bellied capuchin (*Sapajus xanthosternos*)	Brazil		[5]
Black-capped squirrel monkey (*Saimiri boliviensis*)	Amazon basin in Bolivia, western Brazil, and eastern Peru		[5]
Common squirrel monkey (*Saimiri sciureus*)	Brazil, Colombia, Ecuador, French Guiana, Guyana, Peru, Suriname, Venezuela	JX045641	[12]
Bare-eared squirrel monkey (*Saimiri ustus*)	Brazil, Bolivia	KC906728	[9]

**Unpublished: Bueno et al.

### Genomic studies of *Plasmodium brasilianum*

*Plasmodium brasilianum* is a parasite thought to be closely related to *P. malariae*, and blood-stage infections of the two species present a morphologically identical picture, with discrimination determined by the host, monkey or human, respectively. The few molecular epidemiological studies reported so far have shown that *P. brasilianum* and *P. malariae* infections are almost indistinguishable genetically. Sequencing studies of the gene coding for the circumsporozoite protein (csp) appear not to differentiate the identity of the two parasites [14–16]. Similar, studies involving the merozoite surface protein-1 (msp1), the ssrRNA small subunit (18S) of ribosomes and the mitochondrial gene cytochrome b (cytb), have identified sequences that were 100% identical or that had only a few randomly distributed single nucleotide position differences [7, 13, 15–18]. Further, the close genetic resemblance of these parasites has been observed across studies in Brazil, Venezuela, Costa Rica, Peru, Colombia and French Guiana from infected humans, monkeys and mosquitoes [7–9, 11, 12, 15–18]. Under conditions of close contact, as shown in Yanomami people and monkeys species in the Venezuelan Amazon, both humans and non-human primates shared quartan parasites without any host specificity that are genetically identical in target candidate genes [13].

A small study using microsatellite genotyping showed that in 14 *P. malariae* isolates from infected individuals from the Brazilian Atlantic forest, all isolates had identical haplotypes, while in one mosquito sample from the same region a different haplotype was found [19]. In the same study, three *P. brasilianum* isolates from non-human primates sampled from a different region (Amazonia) were analysed, and diverse haplotypes were observed. Unfortunately, across all such studies to date only a small number of samples have been compared at only a few genetic loci. To understand the degree of similarity among *P. brasilianum* and *P. malariae* parasites, a comprehensive analysis of whole genome sequencing data is necessary, using many more parasites obtained from different hosts, across a range of geographic regions. Only one draft reference genome of *P. brasilianum* is available [20]. Similarly, only a few genomes are available for *P. malariae*, sourced from Africa and Asia, and none from South America [8, 20–22]. The apicoplast and mitochondrion genomes of *P. brasilianum* are indistinguishable from those of the *P. malariae* reference genome [20, 23], but further comparative analysis of nuclear genomes is needed to fully understand the status of these two species. This is made difficult by the scarcity of whole genome data, so it remains an open question whether these parasites are variants of a single species that is naturally adapted to both human and New World monkey hosts, and freely circulates between them. Related to this, it is also difficult to infer the direction of the cross-species transfer. Nevertheless, the similarity of these parasites suggests that monkeys can act as reservoirs of *P. malariae* / *P. brasilianum*, and this must be considered in control and eradication programmes.

## *Plasmodium malariae*

### History & discovery; epidemiology and disease

As Collins and Jeffery relate [24], *P. malariae* was named by Grassi and Feletti in 1890, following the observations of Golgi in 1886, who noted the existence of malaria parasites with either 48 h or 72 h cycles of fever, the latter subsequently being recognized as characteristic of *P. malariae* infections. This slow-growing species is widely distributed across the tropics and sub-tropics, with often asymptomatic infections characterized by low parasitaemia and a recognized ability to persist in a single host for years or decades [25, 26]. There is evidence that *P. malariae* can survive combination therapies used for treating acute *P. falciparum* malaria, and may present as a post-treatment recrudescence in *P. falciparum* patients [27–29]. Clinical malaria caused by *P. malariae* rarely progresses to severe, complicated or life-threatening illness, although the literature contains consistent reports of mortality due specifically to either glomerulonephritis or severe anaemia in small children with chronic infections [30].

### Distribution and abundance

**Fig. 1:**
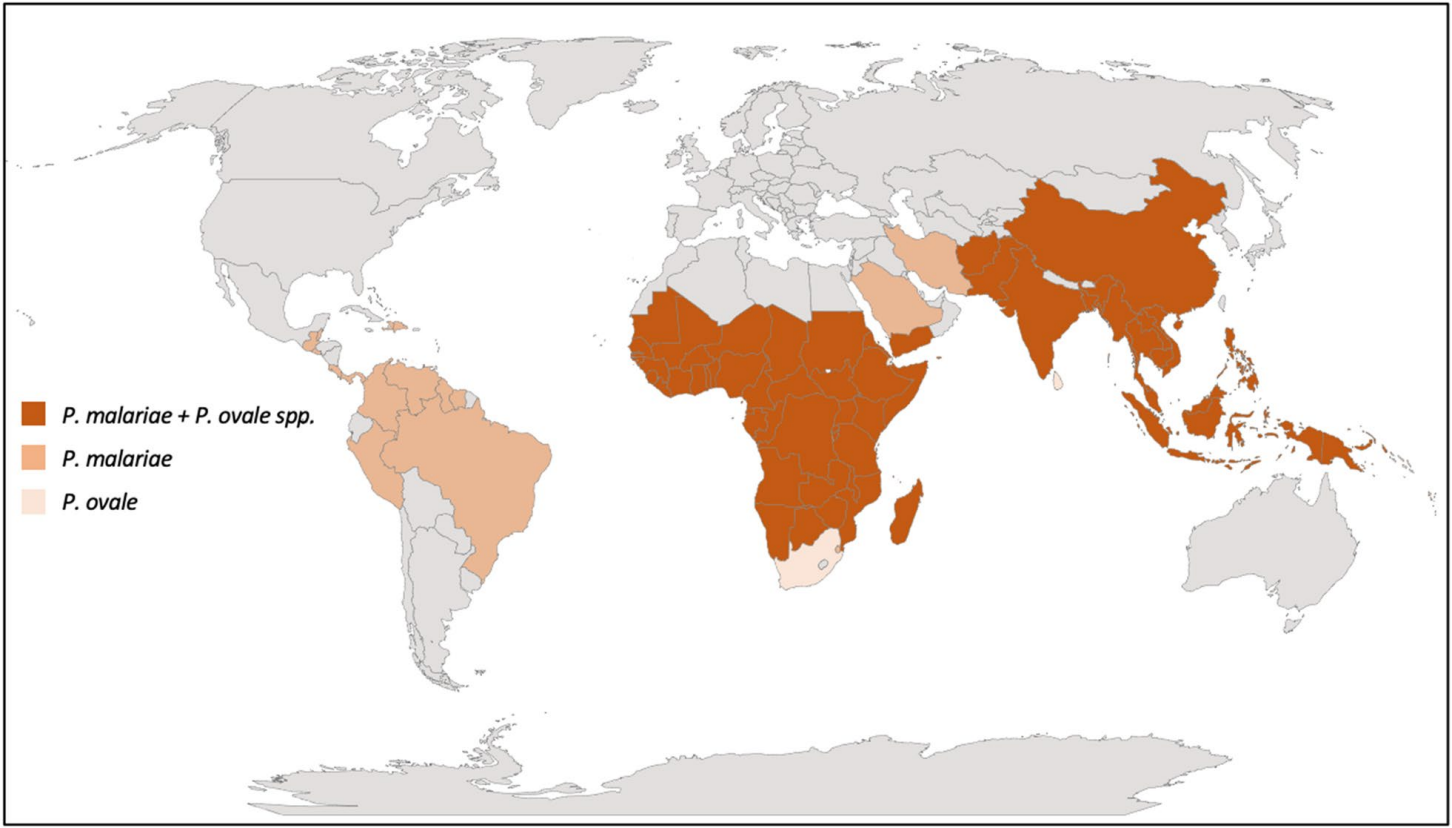
Reported global distributions of *P. malariae* and *P. ovale* spp.

*Plasmodium malariae* is a cosmopolitan parasite distributed in sub-Saharan Africa, South-East Asia, western Pacific islands, and Central and South America [24]. Formerly this parasite was also present in the southern parts of the USA, Argentina, Bhutan, Brunei, South Korea, Morocco, Turkey, and parts of Europe where malaria was eradicated [31–33]. The distribution of this parasite is variable and patchy, and limited to particular mosquito vectors (sporogony needs a minimal temperature of 15 °C), yet autochthonous *P. malariae* cases have been documented from much of the tropics and sub-tropics (Fig. 1; Table 2) [34–143].

**Table 2:** Geographic distribution and prevalence of *P. malariae*

**Country**	**Region**	**Diagnostic Technique**	**Prevalence**	**References**	
Afghanistan	Jalalabad	PCR	0.3% (1/306)	Mikhail et al. 2011	[34]
	Laghman District	Microscopy	1 case	Ramachandra 1951	[35]
	Chardhi	Microscopy	1.4% (1/71 infants)	Ramachandra 1951	[35]
Angola	Bengo povince	PCR	8.1% of malaria positives; 1.3% general	Fancony et al. 2012	[36]
	Luanda	PCR	1.2% (1/81 symptomatic)	Pembele et al. 2015	[37]
Bangladesh	Bandarban	PCR	2.7% (60/2246); 8% of 746 malaria positives; 4.3% of symptomatic patients	Fuehrer et al. 2014	[38]
Belize		MoH official data	0.04% of malaria positives (1990–2008)	Bardach et al. 2015	[31]
Benin		PCR	8.3% (12/144)	Doderer-Lang et al. 2014	[39]
Botswana	Tutume	PCR	0.6% (2/320 asymptomatic)	Motshoge et al. 2016	[40]
	Francistown	PCR	0.5% (1/195 asymptomatic)	Motshoge et al. 2016	[40]
	Kweneng East	PCR	0.4% (3/687 asymptomatic)	Motshoge et al. 2016	[40]
Brazil		MoH official data	0.08% (1990–2008)	Bardach et al. 2015	[31]
	Apiacás—Mato Grosso State	PCR	11.9% (59/497)	Scopel et al. 2004	[41]
	Amazon Region	PCR	33.3% (42/126 malaria positives)	Cunha et al. 2021	[42]
	Espírito Santo	PCR	2.3% (2/92)	de Alencar et al. 2018	[43]
Burkina Faso		PCR	0.1% (1/695 pregnant)	Williams et al. 2016	[44]
	Kossi District	PCR	2.1–13.4% prevalence (decreasing from 2000–2011)	Geiger et al. 2013	[45]
	Bassy and Zanga	PCR	7.4% (8/108) of Pf positives	Culleton et al. 2008	[46]
	Laye	Microscopy	0.9–13.2% (children)	Gnémé et al. 2013	[47]
Burma/Myanmar	Kachin State	PCR	0.1% (3/2598)	Li et al. 2016	[48]
	northern Myanmar	Microscopy	0.04 (2/5585)	Wang et al. 2014	[49]
Burundi	Karuzi	Microscopy	6.7% (228/3393)	Protopopoff et al. 2008	[50]
	Northern Imbo Plain	Microscopy	5% (23/459 malaria positives)	Nimpaye et al. 2020	[51]
Cambodia		PCR	–	Khim et al. 2012	[52]
	Ratanakiri	PCR	2.1% (33/1792)	Durnez et al. 2018	[53]
	2007 Cambodian National Malaria Survey	PCR	0.2% (17/7707)	Lek et al. 2016	[54]
Cameroon		PCR	–	Khim et al. 2012	[52]
	Yaoundé region	PCR	–	Tahar et al. 1998	[55]
	Adamawa region	PCR	17.7% (of 1367)	Feufack-Donfack et al. 2021	[56]
	Yaoundé region	PCR	12% (of 122 asymptomatic children)	Roman et al. 2018	[57]
Central African Republic	Dzanga-Sangha Protected Area	PCR	0.2% (2/95 asymptomatic)	Mapua et al. 2018	[58]
	Dzanga-Sangha region	PCR	11.1% (of 540 symptomatic)	Bylicka-Szczepanowska et al. 2021	[59]
Chad		Microscopy	1 case (infant; mixed with Pf)—imported case in the Netherlands	Terveer et al. 2016	[60]
China	Yunnan	PCR	1% (1/103)	Li et al. 2016	[48]
Colombia	Colombia’s Amazon department	PCR	38.65% (of 1392 symptomatic)	Nino et al. 2016	[61]
		MoH official data	0.03% (1990–2008)	Bardach et al. 2015	[31]
	Colombian Amazon trapezium	PCR	43.2% (862/1995 symptomatic)	Camargo et al. 2018	[62]
Comores	Grande Comore	PCR	0.62% (1/159)	Papa Mze et al. 2016	[63]
Congo DRC	Kinshasa province	PCR	39% asymptomatic and 7% symptomatic (of malaria positives)	Nundu et al. 2021	[64]
		PCR	3.7% (mixed with Pf of malaria positives)	Kiyonga Aimeé et al. 2020	[65]
		PCR	1.5% (1/65; mixed with Pf; asymptomatic children)	Podgorski et al. 2020	[66]
		PCR	4.9% (7/142; 6 mixed with Pf; symptomatic)	Kavunga-Membo et al. 2018	[67]
Congo Republic		PCR	0.9% (8 of 851)	Culleton et al. 2008	[46]
Costa Rica		PCR	4 cases	Calvo et al. 2015	[68]
Cote d'Ivoire		PCR		Khim et al. 2012	[52]
	Yamoussoukro	PCR	1.6% (7/438) febrile; 2.3% (8/346) afebrile	Ehounoud et al. 2021	[69]
Dominican Republic		MoH official data	0.02% (1990–2008)	Bardach et al. 2015	[31]
El Salvador		MoH official data	0.01% of malaria positives (1990–2008); free of malaria since 2021	Bardach et al. 2015	[31]
Equatorial Guinea	Bioko Island (Ureka, Bareso, Sacriba)	PCR	10–31% (asymptomatic < 10 years)	Guerra-Neira et al. 2006	[70]
	Bioko Island	PCR	15.3% (9/59; blood donors)	Schindler et al. 2019	[71]
Eritrea	Eritrean migrants		0.7% (of 146)	Schlagenhauf et al. 2018	[72]
Ethiopia	Southern Ethiopia Omo Nada	PCR	2 mono and 2 mixed with Pf	Mekonnen et al. 2014	[73]
	Amhara Regional State	PCR	0.3% (1/359)	Getnet et al. 2015	[74]
French Guyana		MoH official data	1.39% of malaria positives (1990–2008)	Bardach et al. 2015	[31]
		PCR	Case (GenBank: AF138881)	Fandeur et al. 2000	[7]
Gabon	Franceville	PCR	2.5% (4/162); febrile children	Maghendji-Nzondo et al. 2016	[75]
	Lambarene	PCR	0.5% (1/206)	Culleton et al. 2008	[46]
	Fougamou and villages in the surroundings	PCR	23% (193/834)	Woldearegai et al. 2019	[76]
Gambia		Microscopy	rarely	http://www.rollbackmalaria.org/files/files/countries/Gambia.pdf (accessed: July 25^th^, 2017)	
Ghana	Kwahu-South	PCR	12.7% (18/142)	Owusu et al. 2017	[77]
		PCR	12.8% (45/352) coinfections with Pf	Culleton et al. 2008	[46]
	Ahafo Ano South District of the Ashanti region	PCR	28% (76/274) school children	Dinko et al. 2013	[27]
Guatemala		MoH official data	0.01% of malaria positives (1990–2008)	Bardach et al. 2015	[31]
Guinea		PCR		Khim et al. 2012	[52]
		Microscopy	0.3% (2/724) in young infants, 12.0% (90/748) in children 1–9 years of age, and 5.8% (43/743) in children 10–15y. 97% (131/135) mixed with Pf	Ceesay et al. 2015	[78]
Guinea-Bissau		PCR		Tanomsing et al. 2007	[79]
	Antula	PCR	18% (of 60) in 1995; 4% (of 71) in 1996	Arez et al. 2003	[80]
Guyana	Georgetown	PCR	3 PCR confirmed cases	Baird et al. 2002	[81]
		MoH official data	0.03% of malaria positives (1990–2008)	Bardach et al. 2015	[31]
Haiti		PCR	Imported to Jamaica	Lindo et al. 2007	[82]
India		PCR	GenBank ID: KU510228	Krishna et al. unpublished	
		various	rare	Reviewed in Chatuverdi et al. 2020	[83]
	Odisha	PCR	9.1% (10/110) mono; 10.9% (12/110) mixed; febrile malaria positives	Pati et al. 2017	[84]
Indonesia	Papua	PCR		Tanomsing et al. 2007	[79]
	Flores—Ende District	PCR	1.9% (of 1509)	Kaisar et al. 2013	[85]
	North Sumatra	PCR	3.4% of 3731 participants; 2.9–11.5% of malaria positives	Lubis et al. 2017	[29]
Iran	Baluchestan	PCR	1.4% (2/140)	Adel and Ashgar 2008	[86]
Kenya	Lake Victoria basin Western Kenya	PCR	5.3% (35/663) of asymptomatic infections and 3.3% (8/245) of clinical cases	Lo et al. 2017	[87]
	Kisii district	PCR	11.6% (84 of 722)	Culleton et al. 2008	[46]
Laos		PCR		Tanomsing et al. 2007	[79]
	northern provinces	PCR	0.05% (3/5082); 7.7% of PCR positives for malaria; 2 mono + 1 mixed Pv	Lover et al. 2018	[88]
Liberia	Far	microscopy	39%	Björkman et al. 1985	[89]
		PCR	3 cases imported to China	Cao et al. 2016	[90]
Madagascar		PCR		Khim et al. 2012	[54]
	Ampasimpotsy	PCR	2.1% (12/559 malaria positives)	Mehlotra et al. 2019	[91]
Malawi		PCR	1 case imported to China	Cao et al. 2016	[90]
	Dedza and Mangochi	PCR	9.4% of 2918	Bruce et al. 2011	[92]
Malaysia	Malaysian Borneo	PCR	2.8% (1/47)	Lee et al. 2009	[93]
	Sabah	PCR	0.6% (8/1366); 7 mono + 1 mixed with Pf	William et al. 2014	[94]
	Peninsular Malaysia	PCR	18% (20/111) of malaria positives; 16 mono; 1 with Pf and 3 with Pk	Vythilingam et al. 2008	[95]
Mali		PCR		Khim et al. 2012	[52]
		PCR	14/603; 3 mono, 10 Pf mix, 1 Pf, PoC mix; pregnant	Williams et al. 2016	[44]
	Northern Mali	PCR	9.4–22.5% of malaria positives—asymptomatic	Koita et al. 2005	[96]
Mauritania	Boghe-Sahelian zone	Microscopy	0.03% (1/3445 children); 0.7% (1/143 malaria positives)	Ouldabdallahi Moukah et al. 2016	[97]
	Hodh Elgharbi (Sahelian zone)	Microscopy	1.1% (4/378) of malaria positives febrile patients; 0.3% (4/1161) in febrile patiens	Ould Ahmedou Salem et al. 2016	[98]
Mayotte	Mayotte Island	Microscopy	4% of all malaria positive cases	Maillard et al. 2015	[99]
Mozambique	Manchiana and Ilha Josina	PCR	Manchiana: 19.3% (27/140); Ilha Josina: 28.7% (54/188)	Marques et al. 2005	[100]
Namibia	Bushmanland	Microscopy	rare	mentioned in Noor et al. 2013	[101]
Niger	south-eastern	Microscopy	1.7% of malaria positves	Doudou et al. 2012	[102]
Nigeria	Ibadan area	PCR	11.7% (69/590), children; mainly mixed infections	May et al. 1999	[103]
	Eboyi State	PCR	6.67% mono; 2% mixed with pf of 150 HIV positive patients	Nnoso et al. 2015	[104]
	Lafia	PCR	0.7% (7/960)—3 mono and 4 mixed Pf, asymptomatic children	Oyedeji et al. 2017	[105]
	Ibadan	PCR	66% (352/530) of malaria positive asymptomatic adolescents (ages 10–19 years), mainly mixed	Abdulraheem et al. 2021	[106]
Pakistan		PCR	1 case imported to China	Cao et al. 2016	[90]
		Microscopy	0.4% (2/521) hospitalized patients	Beg et al. 2008	[107]
Panama		MoH official data	0.01% of malaria positives (1990–2008)	Bardach et al. 2015	[31]
			Eradicated?—Last case in 1972	Hurtado et al. 2020	[108]
Papua New Guinea	East Sepik Province	PCR	4.62% (100/2162); 75 mono and 25 mixed	Mehlotra et al. 2000	[109]
		PCR	Oro (0.7%); Eastern Highlands (0.2%); Madang (1.5%); New Ireland (1.3%); East New Britain (0.3%); Bougainville (0.1%)	Hetzel et al. 2015	[110]
Peru	south-east Amerindian population	microscopy	above 80% of all malaria infections	Sulzer et al. 1975	[111]
		MoH official data	0.02% of malaria positives (1990–2008)	Bardach et al. 2015	[31]
Philippines	Palawan	Microscopy	0–0.5%	Oberst et al. 1988	[112]
	Mindanao	PCR	0.03% (1/2639) asymptomatic	Dacuma et al. 2021	[113]
Rwanda	Rukara Health Centre	PCR	1% (1/99)	Culleton et al. 2008	[46]
Sao Tome/Principe	Principe	Microscopy	11 cases	Lee et al. 2010	[114]
Saudi Arabia	Western regions	Microscopy	0.5% (48/8925 malaria positives)	Amer et al. 2020	[115]
Senegal	Kedougou	PCR	GenBank ID: KX417705	unpublished	
	southeastern Senegal	PCR	3.3% of 122 asymptomatic participants	Badiane et al. 2021	[116]
Sierra-Leone	Moyamba District	Microscopy	2.1% Pm mono	Gbakima et al. 1994	[117]
	Bo	PCR	0.4% (2/534) febrile patients	Leski et al. 2020	[118]
Somalia		microscopy	5% of all malaria positives	reviewed in Oldfield et al. 1993	[119]
	Imported to USA—marines	microscopy	0.9% (1/106)	Newton et al. 1994	[120]
South Sudan	Jonglei State	microscopy	6 of 392; 7.7% of malaria positives	Omer et al. 1978	[121]
Sudan	Gezira	microscopy	38 of 1987; 4.1% of malaria positives	Omer et al. 1978	[121]
	East Sudan	PCR	case report	Imirzalioglu et al. 2006	[122]
	Red Sea State	microscopy	1.1% (3/283 malaria positives)	Ageep 2013	[123]
Suriname		MoH official data	5.25% of malaria positives (1990–2008)	Bardach et al. 2015	[31]
		microscopy	12% of 86 Pf positives	Peek et al. 2004	[124]
Swaziland		PCR	0.02% (1/4028)	Hsiang et al.2012	[125]
Tanzania	Zanzibar	PCR	24—14 mono and 10 mixed Pf	Xu et al. 2015	[126]
	Zanzibar	PCR	0.5% (3/594) febrile patients but Pf-RDT negative	Baltzell et al. 2013	[127]
	Kibiti District	PCR	2.4% in 2016 (11.3–16.2% in the 1990’s)	Yman et al. 2019	[128]
Thailand		PCR	Various GenBank entries (e.g. EF206337)	Tanomsing et al. 2007	[79]
	Kanchanaburi Province	PCR	0.2% (2/812)	Yorsaeng et al. 2019	[129]
		MoH	2012: 0.3% (48/16196 malaria positives) 2013: 0.5% (80/14740 malaria positives) 2015: 0.2% (26/12637 malaria positives) 2016: 0.2% (26/15451 malaria positives)	Summarized in Yorsaeng et al. 2019	[129]
Timor-Leste		Microscopy	0.57% (6 cases)	Bragonier et al. 2002	[130]
	Imported to Australia		0.6% (3/501 malaria positives from East Timor; 1 mono and 2 mixed)	Elmes 2010	[131]
Togo		PCR		Khim et al. 2012	[52]
		microscopy		Dorkenoo et al. 2016	[132]
Uganda		PCR	GenBank ID:AB354570	Hayakawa et al. 2008	[133]
		PCR	4.8% (48/1000) blood donors; 31.2% of all malaria positives	Murphy et al. 2020	[134]
Vanuatu				Mentioned in Maguire et al. 2006	[135]
Venezuela		PCR	Various; e.g. KM016331	Lalremruata et al. 2015	[13]
	Yanomami villages	PCR	11.8% (75/630); 25 mixed infections	Lalremruata et al. 2015	[13]
		MoH official data	0.09% of malaria positives (1990–2008)	Bardach et al. 2015	[31]
Vietnam		PCR	Various GenBank entries (e.g. EF206329)	Tanomsing et al. 2007	[79]
	Khanh Hoa Province	PCR	4.8% (6/125) malaria positives	Maeno et al. 2017	[136]
	Ninh Thuan Province	PCR	30.4% (204/671) of malaria positives; 95 mono and 109 mixed infections	Nguyen et al. 2012	[137]
Yemen	Taiz-region	Microscopy	0.06% (1/1638) asymptomatic	Al-Eryani et al. 2016	[138]
	highlands	Microscopy	0.2% (1/455) symptomatic; 1.3% (1/78) *Plasmodium* positives	Al-Mekhlafi et al. 2011	[139]
Zambia	Nchelenge District	Microscopy	0.6% (5/782) Children < 10 years; 2.1%, (5/236) of malaria positives	Nambozi et al. 2014	[140]
	Western and Southern Province	PCR	1.7% (5/304); 2 mono and 3 mixed Pf	Sitali et al. 2019	[141]
	Choma District, Southern Province	PCR	0.2% of 3292 participants; 2 Pm and 5 Pm + Pf; low transmission area	Laban et al. 2015	[142]
Zimbabwe		Microscopy	1.8% of 51,962; 8.3% of malaria infections (1972–1981)	Taylor and Mutambu 1986	[143]

Assessment of the abundance of *P. malariae* is difficult because this parasite has been neglected by researchers, and studies differ (e.g. symptomatic patients vs. population studies; Table 2). Some epidemiological studies reported a high prevalence (15–30%) in Africa, Papua New Guinea, and the Western Pacific, in contrast to scanty observations (1–2%) from Asia, the Middle East, Central and Southern America [144]. However, with the advent of molecular diagnostic techniques this parasite species has been reported more frequently, being found in regions where it was not previously thought be present (e.g. Bangladesh), more commonly observed in mixed infections with *P. falciparum* [24], and identified as recrudescent infections in historical cases from areas such as Greece, formerly endemic for malariae malaria, but since having eliminated contemporary transmission of the disease [145].

### Genomic studies of *Plasmodium malariae*

Large-scale genomic studies of the neglected malaria parasites and zoonotic species have been difficult to date, limited by infections having low parasite densities and being mixed with other *Plasmodium* species, thereby making it difficult to obtain sufficient parasite DNA to perform whole genome sequencing. For *P. malariae*, the first partial genome using next-generation sequencing was produced from CDC Uganda I strain DNA [22, 146]. A subsequent study generated a more complete reference using long-read sequencing technology from DNA of the *P. malariae* isolate PmUG01, from an Australian traveller infected in Uganda [22, 23]. Additional genomic data from short-read Illumina data of travellers’ isolates from Mali, Indonesia and Guinea, and one patient in Sabah, Malaysia, were also reported by Rutledge et al*.* Analysis of these genomes revealed that around 40% of the 33.6 Mbp genome (24% GC content), particularly in subtelomeric chromosome regions, is taken up by multigene families, as seen in *P. ovale* species [22, 25]. The *P. malariae* genome displays some unique characteristics, such as the presence of two large families, the *fam-l* and *fam-m* genes, with almost 700 members [22, 23]. Most of these genes encode proteins with a PEXEL export signal peptide and many encode proteins with structural homology to Rh5 of *P. falciparum*, the only known protein that is essential for *P. falciparum* red blood cell invasion [147]. These observations suggest that the *fam-l* and *fam-m* gene products may also have an important role in binding to host ligands. Other gene families, such as the *Plasmodium* interspersed repeat (*pir*) loci that are present in many species in the genus, including in *Plasmodium vivax* (~ 1500 *vir* genes), are present in the *P. malariae* genome. Of the 250 *mir* genes identified, half are possible pseudogenes. Products of the *pir* genes are predicted to be exported to the infected erythrocyte surface and may have a role in cell adhesion. Like *pir* genes, SURFIN proteins are also encoded in the *P. malariae* genome at around 125 loci, much greater than the number present in *P. falciparum* (ten) or *P. vivax* (two). Another unique feature of the *P. malariae* genome is the presence of 20 copies, in a single tandem array, of the P27/25 gene, a sexual-stage cytoplasmic protein with a possible role in maintaining cell integrity. P27/25 is encoded by a single copy gene in all other species evaluated to date [23, 25].

The sequences of an additional eighteen *P. malariae* genomes from Africa and Asia have recently been reported [21]. These were derived directly from patient isolates, using a selective whole genome DNA amplification (SWGA) approach to increase the relative abundance of parasite DNA sequence reads relative to host reads. A total of 868,476 genome-wide SNPs were identified, filtered to 104,583 SNPs after exclusion of the hypervariable subtelomeric regions. Phylogenetic analysis showed a clear separation of isolates sourced from Africa and Asia, similar to observations from the analysis of sequence data from the circumsporozoite (*pmcsp*) gene [148]. Many non-synonymous SNPs in orthologs of *P. falciparum* drug resistance-associated loci (*pmdhfr*, *pmdhps* and *pmmdr1*) were detected [21, 52], but their impact on drug efficacy remains unknown. Thus, to date, there are no validated molecular markers of drug resistance in *P. malariae* parasites although, as noted above, prophylaxis breakthrough, treatment failures and emergence following treatment for other species have been reported [26–29, 149].

In the wider *Plasmodium* species context, phylogenetic analysis has shown that *P. malariae* isolates group with malariae-like species that infect monkeys and non-human primates [2, 23]. *Plasmodium malariae* parasites also cluster closer to *P. ovale* spp., but in separate clades, and more generally in a clade with *P. vivax*, *P. knowlesi* and *Plasmodium cynomolgi* that is distant from the Laverania sub-genus exemplified by *P. falciparum* and *Plasmodium reichenowi* [2, 150]. Given the range of primate hosts that are infected by *P. malariae, P. brasilianum* and their close relatives, further genomic studies are needed to tease out the two main questions raised by the studies so far:

o Should *P. brasilianum*, as is currently circulating in South America, and *P. malariae* be considered distinct, non-recombining species?
o What is the extent of the radiation of *P. malariae-*like species in the great apes?

## *Plasmodium ovale curtisi* and *Plasmodium ovale wallikeri*

### History & discovery

First identified in Liverpool by Stephens in 1918, the index case of ovale malaria was a British army private, returning to the UK in 1918 following deployment in “East Africa”, and having reported an episode of symptomatic malaria in December, 1916 [151]. This soldier’s blood films were examined over several months, with no mention of any treatment being offered, during which time the presence of fimbriated, oval infected red cells was noted as a key feature, together with a 48 h fever periodicity. This “new parasite of man” (sic) was thus characterized as a benign tertian infection and named *Plasmodium ovale* in the primary paper, published in 1922*.* Some additional detailed description of the parasite and its presentation was published by Stephens and Owen in 1927 [152].

For much of the twentieth century, ovale malaria remained a minor entrant in parasitology textbooks, including Coatney et al. [1], until the advent of molecular diagnostic studies in the 1990s began to uncover evidence of genetic dimorphism [153], leading to a series of papers in the first decade of the twenty-first century examining the impact of this dimorphism on molecular and antigen-based diagnosis [154–158]. A multi-centre effort to gather 51 geographically diverse parasite isolates and generate sequencing data across seven genetic loci was then able to demonstrate that ovale malaria was the result of infection by either of two non-recombining, sympatric sibling parasite species, which were named *P. ovale curtisi* and *P. ovale wallikeri* [159]. In the decade that followed, various molecular tools were developed to distinguish the two ovale species, and there was an explosion of our understanding of the contribution of the newly recognized parasites to malaria burden across the tropics.

### Distribution and abundance

Although the original identification of *P. ovale *sensu lato (*s.l.*) by Stephens was in a British soldier who contracted malaria in “East Africa”, the species was subsequently recognized as highly endemic in West Africa (especially Nigeria). Coatney et al. described the distribution of the species as extending to the East African Coast, and as far south as Mozambique [1]. Outside Africa, ovale malaria was sporadically reported from Papua New Guinea, Indonesian islands and some South-East Asian countries [144]. However, with the introduction of molecular diagnostic tools and recognition and widespread acceptance of the two sympatric species, *P. o. curtisi* (former “classic” type) and *P. o. wallikeri* (former “variant” type) [159], a much more complex understanding of these parasites has developed. Molecular diagnostics have greatly facilitated the confirmation of the presence of ovale malaria parasites in much of Africa and Asia, including countries where it was not previously known to be present (e.g. Bangladesh, Afghanistan, Angola) [35–37, 160–162], and in non-human primates [163]. However, it remains generally accepted that these parasites are not endemic in the Americas [159].

**Fig. 2:**
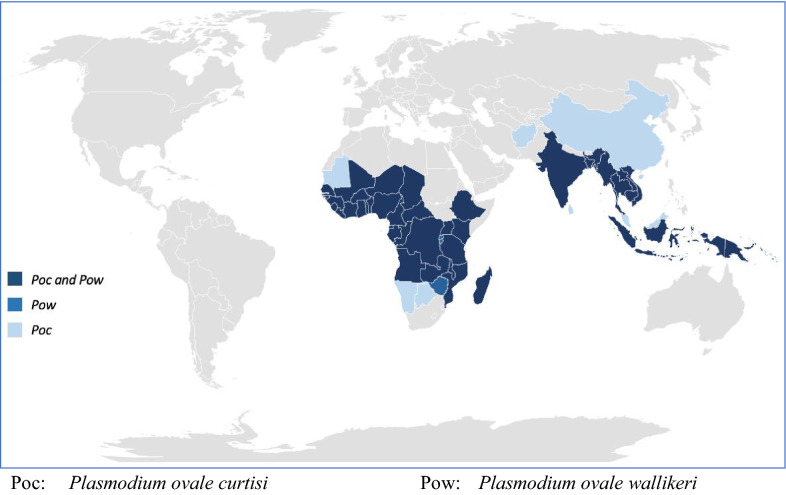
Reported global distributions of *P. ovale curtisi* and *P. ovale wallikeri. Poc Plasmodium ovale curtisi, Pow Plasmodium ovale wallikeri*

Infections with ovale malaria parasites are often asymptomatic and parasite densities low, leading to difficulties in accurate microscopic diagnosis and some uncertainties as to distribution in the recent past. Given the presence of intra-erythrocytic stippling on thin films, and the irregular shapes adopted by ovale-infected cells, there is some morphological similarity to *P. vivax,* which exacerbates diagnostic difficulties. This also influenced early phylogenetic thinking; Coatney and colleagues write that “from the vivax-like stem developed a morphologically similar species, *P. ovale*, that was capable of surviving in (African) hominids …” (1). Moreover, mixed infections with other human malaria parasites are very common. Double infections of *P. ovale curtisi* and *P. ovale wallikeri* in the same individual have also been reported (e.g. Angola, Bangladesh) [36, 161], confirming the lack of recombination between the two species. However, reported prevalence estimates vary widely among various studies, reflecting different study designs and blood sample collection strategies (e.g. asymptomatic vs. febrile patients). The known distribution of *P. ovale* spp., *P. o. wallikeri* and *P. o. curtisi* is presented in Fig. 2, and a detailed listing of reports identifying these species, including GenBank accession ID where relevant, is given in Table 3 [27, 36, 48, 58, 72, 76, 83, 90, 97, 102, 106, 116, 118, 137, 156, 159, 166–217].

**Table 3:** Geographic distribution and prevalence of *P. ovale* sp., *P. ovale wallikeri* and *P. ovale curtisi* (Sequences submitted to GenBank as *P. ovale* were assigned to species level post hoc)

**Country**	**Type**	**Diagnostic Technique**	**Prevalence**	**References**	
Afghanistan	*P. ovale curtisi*	PCR	Imported to Switzerland	Nguyen et al. 2020	[162]
Angola	*P. ovale curtisi*	Sequence	GenBank: FJ409571; FJ409567	Duval et al. 2009	[163]
	*P. ovale wallikeri*	Sequence	GenBank: MG588149; imported to China	Zhou et al. Unpublished	–
	*P. ovale wallikeri*	PCR	0.3% (11/3316) 3 mono + 8 mixed; 2% (11/541) malaria positives	Fançony et al. 2013	[36]
	*P. ovale curtisi*	PCR	0.3% (11/3316) 4 mono + 7 mixed; 2% (11/541) malaria positives	Fançony et al. 2013	[36]
Bangladesh	*P. ovale curtisi*	Sequence	0.26% (1/379) symptomatic; 0.45% (10/1867) incl. asymptomatic participants; Mono—36.4%	Fuehrer et al. 2012	[161]
	*P. ovale wallikeri*	Sequence	0.79% (3/379) symptomatic; 0.53% (12/1867) incl. asymptomatic participants; Mono—46.1%	Fuehrer et al. 2012	[161]
Benin	*P. ovale wallikeri*	Sequence	GenBank: GQ183063; EU266604	Sutherland et al. 2010	[159]
	*P. ovale wallikeri*	PCR	1 isolate in meta-analysis	Bauffe et al. 2012	[164]
	*P. ovale curtisi*	PCR	2 isolates in meta-analysis	Bauffe et al. 2012	[164]
Botswana	*P. ovale curtisi*	PCR	1.85% (30/1614); 11 mono and 19 mixed	Motshoge et al. 2021	[165]
Brunei	*P. ovale* sp.		1 case imported to China	Cao et al. 2016	[90]
Burkina Faso	*P. ovale curtisi*	PCR	3 isolates	Calderaro et al. 2012	[166]
	*P. ovale wallikeri*	PCR	Imported to Germany	Frickmann et al. 2019	[167]
Burma/Myanmar	*P. ovale curtisi*	Sequence	Various: e.g. KX672039; AB182496	Win et al. 2004; Li et al. 2016	[48, 156]
	*P. ovale wallikeri*	Sequence	Various: e.g. AB182497	Win et al. 2004	[48]
Burundi	*P. ovale wallikeri*	PCR	1 isolate, imported to UK	Nolder et al. 2013	[168]
Cambodia	*P. ovale curtisi*	Sequence	GenBank: e.g. FJ409571	Duval et al. 2009	[163]
	*P. ovale wallikeri*	Sequence		Incardona et al. 2005	[169]
Cameroon	*P. ovale curtisi*	Sequence	Imported to Singapore; GenBank: e.g. KP050401	Chavatte et al. 2015	[170]
	*P. ovale curtisi*	Sequence		Kojom Foko et al. 2021	[171]
	*P. ovale wallikeri*	Sequence	GenBank: e.g. FJ409566	Duval et al. 2009	[56]
Central African Republic	*P. ovale curtisi*	Sequence	Various GenBank: e.g. FJ409571; KP050465	Duval et al. 2009; Chavatte et al. 2015	[163, 170]
	*P. ovale wallikeri*	Sequence	1.1% (1/95) asymptomatics; 4.3% (1/23) of malaria positives; GenBank: MG241227	Mapua et al. 2018	[58]
Chad	*P. ovale curtisi*	PCR	1 isolate in meta-analysis	Bauffe et al. 2012	[164]
	*P. ovale wallikeri*	PCR	1 isolate in meta-analysis	Bauffe et al. 2012	[164]
	*P. ovale curtisi*	PCR	Imported to China	Zhou et al. 2019	[172]
	*P. ovale wallikeri*	PCR	Imported to China	Zhou et al. 2019	[172]
China (Yunnan)	*P. ovale curtisi*	Sequence	GenBank: KX672045; certified malaria free since 2021	Li et al. 2016	[48]
Comoros	*P. ovale curtisi*	PCR	7 isolates	Bauffe et al. 2012	[164]
	*P. ovale wallikeri*	PCR	11 isolates	Bauffe et al. 2012	[164]
Congo DRC	*P. ovale curtisi*	Sequence	GenBank: e.g. FJ409567	Duval et al. 2009	[163]
	*P. ovale wallikeri*	Sequence	1% (2/198) children < 5 years; GenBank: KT867772	Gabrielli et al. 2016	[173]
Congo Republic of the	*P. ovale curtisi*	Sequence	Imported to China; GenBank: MT430962	Chen et al. 2020	[174]
	*P. ovale curtisi*	PCR	4 clinical cases	Oguike et al. 2011	[175]
	*P. ovale wallikeri*	PCR	2 clinical cases	Oguike et al. 2011	[175]
Cote d’Ivoire	*P. ovale curtisi*	Sequence	GenBank: e.g. FJ409567; KP050411	Duval et al. 2009; Chavatte et al. 2015	[163, 170]
	*P. ovale wallikeri*	Sequence	GenBank: e.g. GU723538	Sutherland et al. 2010	[159]
Djibouti	*P. ovale* sp.		Rarely, 1 case in 2018/19 season	de Santi et al. 2021	[176]
East Timor (Timor-Leste)	*P. ovale* sp.		Present according to WHO; Documented in West Timor	Gundelfinger 1975	[177]
Equatorial Guinea	*P. ovale curtisi*	Sequence	GenBank: JF505386	Unpublished	–
	*P. ovale wallikeri*	Sequence	GenBank: e.g.: KP050469	Chavatte et al. 2015	[170]
	*P. ovale curtisi*	PCR	Bioko Island—0.9–1.4% ovale in total population	Oguike et al. 2011	[175]
	*P. ovale wallikeri*	PCR	Bioko Island—0.9–1.4% ovale in total population	Oguike et al. 2011	[175]
Eritrea	*P. ovale* sp.		1 case—imported to Germany	Roggelin et al. 2016	[178]
	*P. ovale* sp.		2.7% (4/146)—imported to Europe	Schlagenhauf et al. 2018	[72]
Ethiopia	*P. ovale curtisi*	Sequence	0.7% (2/300) of symptomatic patients; 1.1% (2/184) of malaria positives, GenBank: e.g. KF536874	Alemu et al. 2013	[179]
	*P. ovale wallikeri*	Sequence	2.3% (7/300) of symptomatic patients; 3.8% (7/184) of malaria positives, GenBank: e.g. KF536876	Alemu et al. 2013	[179]
Gabon	*P. ovale curtisi*	Sequence	GenBank: e.g.: FJ409571; MG869603	Duval et al. 2009; Groger et al. 2019	[163, 180]
	*P. ovale wallikeri*	Sequence	GenBank: e.g.: KJ170104; MG869598	Groger et al. 2019	[180]
	*P. ovale curtisi*	PCR	Rural Gabon—8.9% of malaria positives; 7 of 74 mono infection	Woldearegai et al. 2019	[76]
	*P. ovale wallikeri*	PCR	Rural Gabon—4.6% of malaria positives; 1 of 38 mono infection	Woldearegai et al. 2019	[76]
Gambia, The	*P. ovale wallikeri*	PCR	0.16% (1/604) pregnant	Williams et al. 2016	[44]
Ghana	*P. ovale curtisi*	Sequence	GenBank: e.g.: GU723554	Sutherland et al. 2010	[159]
	*P. ovale wallikeri*	Sequence	GenBank: e.g.: KP725067	Oguike and Sutherland 2015	[181]
	*P. ovale curtisi*	PCR	Ashanti Region, 4% (15/284) malaria positives	Heinemann et al. 2020	[182]
	*P. ovale wallikeri*	PCR	Ashanti Region, 3% (12/284) malaria positives	Heinemann et al. 2020	[182]
	*P. ovale curtisi*	PCR	27 cases—Children 5–17	Dinko et al. 2013	[27]
	*P. ovale wallikeri*	PCR	7 cases—Children 5–17	Dinko et al. 2013	[27]
Guinea	*P. ovale curtisi*	Sequence	GenBank: e.g.: FJ409571	Duval et al. 2009	[181]
	*P. ovale curtisi*	PCR	Imported to France	Joste et al. 2021	[183]
	*P. ovale wallikeri*	PCR	Imported to China and France	Zhou et al. 2018; Joste et al. 2021	[183, 184]
Guinea-Bissau	*P. ovale curtisi*	Sequence	GenBank: e.g.: EU266611	Sutherland et al. 2010	[159]
	*P. ovale wallikeri*	PCR		Saralamba et al. 2019	[185]
India	*P. ovale curtisi*	Sequence	GenBank: e.g.: KU510234; KP050460	Chavatte et al. 2015; Krishna et al. 2017	[170, 186]
	*P. ovale wallikeri*	Sequence	Mono infection, Bastar division of Chhattisgarh state, GenBank: KM873370	Chaturvedi et al. 2015	[83]
	*P. ovale curtisi*	Sequence	Mono infection, Bastar division of Chhattisgarh state, GenBank: KM288710	Chaturvedi et al. 2015	[83]
Indonesia	*P. ovale curtisi*	Sequence	Sumatra,—GenBank: e.g.: KP050463	Chavatte et al. 2015	[170]
	*P. ovale wallikeri*	Sequence	GenBank: e.g.: AB182497	Win et al. 2004	[167]
Kenya	*P. ovale curtisi*	Sequence	GenBank: e.g.: KM494987	Miller et al. 2015	[186]
	*P. ovale wallikeri*	Sequence	GenBank: e.g.: KM494986	Miller et al. 2015	[186]
Laos	*P. ovale curtisi*	Sequence		Toma et al. 1999	[188]
	*P. ovale wallikeri*	Sequence		Toma et al. 1999	[188]
	*P. ovale* sp.	PCR	0.04% (1/2409) participants	Iwagami et al. 2018	[189]
Liberia	*P. ovale curtisi*	Sequence	GenBank: e.g.: KP050457	Chavatte et al. 2015	[170]
	*P. ovale wallikeri*	Sequence	GenBank: e.g.: KP050382	Chavatte et al. 2015	[170]
Madagascar	*P. ovale curtisi*			Randriamiarinjatovo 2015	[190]
	*P. ovale wallikeri*	Sequence	GenBank: e.g.: FJ409570	Duval et al. 2009	[163]
	*P. ovale* sp.	PCR	1.4% (8/559) of malaria positives; 2 mono infections	Mehlotra et al. 2019	[91]
Malawi	*P. ovale curtisi*	PCR	2 isolates	Oguike and Sutherland 2015	[181]
	*P. ovale wallikeri*	PCR	2 isolates	Oguike and Sutherland 2015	[181]
Malaysia	*P. ovale* sp. (*P. ovale curtisi*)	PCR	0.17% (1/585) asymptomatic; 5.3% (1/19) of malaria positives; primers rOVA1/rOVA2	Noordin et al. 2020	[191]
	*P. ovale curtisi*	Sequence	Pahang; GenBank: MK351321	unpublished	–
	*P. ovale* sp.	PCR	0.4% (2/457) malaria positives	Yusof et al. 2014	[192]
Mali	*P. ovale wallikeri*	Sequence	GenBank: e.g. FJ409566	Duval et al. 2009	[163]
	*P. ovale curtisi*	PCR	0.49% (3/603) in pregnant women; 1 mono + 2 mixed	Williams et al. 2016	[44]
	*P. ovale wallikeri*	PCR	0.49% (3/603) in pregnant women; 3 mixed	Williams et al. 2016	[44]
Mauritania	*P. ovale* sp.	Microscopy	Asymptomatic; Sahelian zone 0.47% (5/1056); Saharan zone 0.18% (2/1059); Sahelo-Saharan zone 0.37% (5/1330)	Ouldabdallahi Moukah et al. 2016	[97]
	*P. ovale curtisi*	PCR	Imported to France	Joste et al. 2021	[183]
Mayotte	*P. ovale* sp.	Regional Health Agency	0.4% of malaria cases	Maillard et al. 2015	[99]
Mozambique	*P. ovale curtisi*	Sequence	GenBank: e.g. GU723517	Sutherland et al. 2010	[159]
	*P. ovale curtisi*	PCR	Imported to China	Cao et al. 2016	[90]
	*P. ovale wallikeri*	PCR	Imported to France and Spain	Rojo-Marcos et al. 2014, Joste et al. 2021	[183, 193]
Namibia	*P. ovale curtisi*	PCR	0.31% (of 952) children < 9 years	Haiyambo et al. 2019	[194]
Niger	*P. ovale* sp.	Microscopy	1 case	Doudou et al. 2012	[102]
	*P. ovale curtisi*	PCR	Imported to France	Joste et al. 2021	[183]
	*P. ovale wallikeri*	PCR	Imported to France	Joste et al. 2021	[183]
Nigeria	*P. ovale curtisi*	Sequence	GenBank: e.g.: GU723534; KP050374	Sutherland et al. 2010; Chavatte et al. 2015	[159, 170]
	*P. ovale wallikeri*	Sequence	GenBank: e.g.: GU723579	Sutherland et al. 2010	[159]
	*P. ovale* sp.	PCR	24% of malaria positives	Abdulraheem et al. 2019	[106]
	*P. ovale curtisi*	PCR	1.1% (4/365) malaria positive children	Oyedeji et al. 2021	[195]
	*P. ovale curtisi*	PCR	Imported to China, France and Spain	Cao et al. 2016; Joste et al. 2021; Rojo-Marcos et al. 2014	[90, 183, 193]
	*P. ovale wallikeri*	PCR	Imported to China, France and Spain	Cao et al. 2016; Joste et al. 2021; Rojo-Marcos et al. 2014	[90, 183, 193]
Pakistan	*P. ovale* sp.	PCR	Imported to China	Cao et al. 2016	[90]
Papua New Guinea	*P. ovale curtisi*	Sequence	GenBank: e.g.: AF145337	Mehlotra et al. 2002	[196]
	*P. ovale wallikeri*	Sequence	GenBank: e.g.: EU266603	Sutherland et al. 2010	[159]
	*P. ovale* sp.	PCR	3.4% of 504 children aged 5–10 y from East Sepik Province	Robinson et al. 2015	[197]
Philippines	*P. ovale* sp.		Rare, Palawan only until 1977	Cabrera and Arambulo 1977	[200]
	*P. ovale* sp.	PCR	Palawan—0.3% (2/613)	Reyes et al. 2021	[199]
Rwanda	*P. ovale wallikeri*	PCR	Imported to France	Joste et al. 2021	[183]
	*P. ovale wallikeri*	Sequence	GenBank: e.g.: FJ409570	Duval et al. 2009	[163]
	*P. ovale* sp.	PCR	4.9% (53/1089) schoolchildren	Sifft et al. 2016	[200]
Sao Tome and Principe	*P. ovale curtisi*	Sequence	GenBank: e.g.: GQ231520	Sutherland et al. 2010	[159]
	*P. ovale wallikeri*	Sequence	GenBank: e.g.: EU266603	Sutherland et al. 2010	[159]
	*P. ovale* sp.	PCR	2.8% of 661	Pinto et al. 2000	[201]
Senegal	*P. ovale curtisi*	Sequence	GenBank: e.g.: KX417703	unpublished	–
	*P. ovale wallikeri*	Sequence	GenBank: e.g.: KX417699	unpublished	–
	*P. ovale* sp.	PCR	4.91% (6/122)	Badiane et al. 2021	[116]
	*P. ovale curtisi*	PCR	Imported to France	Joste et al. 2021	[183]
	*P. ovale wallikeri*	PCR	Imported to France	Joste et al. 2021	[183]
Sierra Leone	*P. ovale curtisi*	Sequence	GenBank: e.g.: GU723523	Sutherland et al. 2010	[159]
	*P. ovale wallikeri*	Sequence	GenBank: e.g.: GU723571	Sutherland et al. 2010	[159]
	*P. ovale curtisi*	PCR	Imported to France	Joste et al. 2021	[183]
	*P. ovale wallikeri*	PCR	Imported to France	Joste et al. 2021	[183]
	*P. ovale* sp.	PCR	0.4% (2/534) febrile patients	Leski et al. 2020	[118]
Solomon Islands	*P. ovale wallikeri*	PCR		Echeverry et al. 2016; Echeverry et al. 2017	[202, 203]
	*P. ovale* sp.	PCR	0.05% (1/1914)	Russell et al. 2021	[204]
Somalia	*P. ovale* sp.		Imported to USA (military)	CDC 1993	[205]
South Africa	*P. ovale* sp.	PCR	Imported to China	Cao et al. 2016	[90]
South Sudan	*P. ovale* sp.	Microscopy	Bor; 1.2% of 392	Omer et al. 1978	[121]
Sri Lanka	*P. ovale curtisi*	PCR	1 isolate in meta-analysis; Sri Lanka malariafree since 2016	Bauffe et al. 2012	[164]
Sudan	*P. ovale* sp.	Microscopy	New Halfa, 2% of 190 malaria positives	Himeidan et al. 2005	[206]
	*P. ovale* sp.	Microscopy	Khartoum; 0.32% of 3791 participants	El Sayed et al. 2000	[207]
	*P. ovale* sp.	PCR	Imported to China	Cao et al. 2016	[90]
Tanzania	*P. ovale curtisi*	Sequence	GenBank: e.g.: GU723515	Sutherland et al. 2010	[159]
	*P. ovale wallikeri*	PCR	1 isolate	Calderaro et al. 2013	[208]
	*P. ovale wallikeri*	PCR	2 cases, Imported to France	Joste et al. 2021	[183]
	*P. ovale* sp.	PCR	Zanzibar; 16.2% (30/185) malaria PCR positives; 10 mono + 20 mixed infections	Cook et al. 2015	[209]
Thailand	*P. ovale curtisi*	Sequence	GenBank: e.g.: KC137349; KF018432	Putaporntip et al. 2013; Tanomsing et al. 2013	[210, 211]
	*P. ovale wallikeri*	Sequence	GenBank: e.g.: GQ231519; KC137344; KF018430	Sutherland et al. 2010; Putaporntip et al. 2013; Tanomsing et al. 2013	[159, 210, 211]
	*P. ovale* sp.	PCR	0.3% (4/1347) asymptomatic participants; 4 mixed infections	Baum et al. 2016	[212]
Togo	*P. ovale* sp.		2.8%	Gbary et al. 1988	[213]
	*P. ovale* sp.		2% of malaria positives	MSPS 2017	[214]
	*P. ovale curtisi*	PCR	12 cases, Imported to France	Joste et al. 2021	[183]
	*P. ovale wallikeri*	PCR	14 cases, Imported to France	Joste et al. 2021	[183]
Uganda	*P. ovale curtisi*	Sequence	GenBank: e.g.: GU723521	Sutherland et al. 2010	[159]
	*P. ovale wallikeri*	Sequence	GenBank: e.g.: GU723573; KP050464	Chavatte et al. 2015; Sutherland et al. 2010	[159, 170]
	*P. ovale curtisi*	PCR	Apac District; Buliisa District; Mayuge District	Oguike et al. 2011	[175]
	*P. ovale wallikeri*	PCR	Apac District; Buliisa District; Mayuge District	Oguike et al. 2011	[175]
	*P. ovale* sp.	PCR	0–6.7% of all malaria; 0–4.3% of population	Oguike et al. 2011	[175]
	*P. ovale* sp.	PCR	Imported to China	Cao et al. 2016	[90]
Vietnam	*P. ovale curtisi*	Sequence	GenBank: e.g.: GU723523	Sutherland et al. 2010	[159]
	*P. ovale wallikeri*	Sequence	GenBank: e.g.: AF387041	Unpublished	–
	*P. ovale* sp.	PCR	0.8% (19/2303) of population	Nguyen et al. 2012	[137]
Yemen	*P. ovale* sp.	Microscopy	1 symptomatic case, Beni-Hussan village	Al-Maktari and Bassiouny 1999	[215]
Zambia	*P. ovale wallikeri*	PCR	1 case	Nolder et al. 2013	[168]
	*P. ovale wallikeri*	LAMP	eastern Zambia	Hayashida et al. 2017	[216]
	*P. ovale curtisi*	LAMP	eastern Zambia	Hayashida et al. 2017	[216]
	*P. ovale* sp.	LAMP	10.6% in asymptomatic participants	Hayashida et al. 2017	[216]
	*P. ovale* sp.	PCR	Western province (cross-sectional survey); 12.4% (32/259); 6 mono + 26 mixed	Sitali et al. 2019	[141]
Zimbabwe	*P. ovale wallikeri*	Sequence	GenBank: e.g.: FJ409570	Duval et al. 2009	[163]
	*P. ovale* sp.		< 2% of malaria positives	Taylor 1985	[217]

### Genomic studies of *P. o. curtisi* and *P. o. wallikeri*

In the period since the two genetically distinct forms of *P. ovale* spp. were recognized, there have been a limited number of studies that have explored the differences between them. A study in UK travellers with ovale malaria by Nolder and colleagues could not identify any robust features of morphology that can distinguish *P. o. curtisi* from *P. o. wallikeri* [168], but were able to provide evidence of a significant difference in the distribution of relapse periodicity: the former species displayed a geometric mean latency of 85.7 days (95% CI 66.1 to 111.1, N = 74), compared to the significantly shorter 40.6 days (95% CI 28.9 to 57.0, N = 60) of the latter. This contrasts with the earlier observation of Chin and Coatney, who conducted studies of experimentally infected volunteers whose initial infections (all with the same “West African strain”) were treated with quinine or chloroquine before extended follow-up for evidence of *P. vivax*-type relapse [218]. These authors concluded that “These results leave little doubt that ovale malaria is a relapsing disease, but there appears to be no definite relapse pattern…” Subsequent studies in European travellers, a group in which super-infection is absent as a potential confounder, have confirmed this difference in latency period between *P. ovale curtisi* and *P. ovale wallikeri* [168, 219, 220]. These studies were also consistent in finding that *P. ovale wallikeri* is associated with low platelet counts and thus more likely to elicit clinical thrombocytopenia, and more likely to be correctly identified by immunochromatographic lateral flow tests that detect the LDH antigen, which fail to identify > 90% of *P. ovale curtisi* infections, a reflection of differences in the amino acid sequence of LDH in the two species [158, 159].

Given the absence of distinguishing morphological characters, despite reliable differences in some clinical and diagnostic features, there has been increasing attention to characterisation of the genomic organisation of the two sibling species as a route to better understanding their divergence from each other, and to describe the level of within-species diversity. Initial efforts were based on direct sequencing of PCR-amplified loci, and gave a general picture of fixed differences in both synonymous and non-synonymous substitutions between the species in almost every coding region examined, but very little intra-species genetic diversity [159–161, 185, 210, 211]. This was also true of genes related to sexual stage development, which had been examined for evidence of a mating barrier between the two species [181]. Whole genome analysis would clearly be very informative, but very few draft genomes of either species are available due to the difficulty in obtaining parasite DNA from these typically very low parasitaemia infections. The first partial genomes to become available were assembled from Illumina short-read sequencing of two isolates of *P. o. wallikeri* from Chinese workers returning from West Africa, as well as one *P. o. curtisi* isolate also from a Chinese worker returning from West Africa and the genome of the chimpanzee-propagated Nigeria I strain [1, 22, 24]. Subsequently, three partial genomes of *P. o. curtisi* from two patients that tested positive for *P. falciparum* in Ghana and one mixed infection from Cameroon, together with two *P. o. wallikeri* genomes obtained from individual patients in Cameroon, were also assembled [23].

Analysis of the *P. ovale* spp. genomes published to date has estimated a total genome length for both species of ~ 35 Mbp (29% GC content), with 40% being subtelomeric [22, 23]. Differences in total length (maximum observed 38Mbp) were observed between isolates, primarily due to differences in the estimated size of expansion of the *ocir/owir* gene families. These species have considerably more *pir* genes (1500–2000), than other human plasmodium parasites (~ 300) [25]. A larger number of *surfin* genes have also been identified, with > 50 present in *P. o. curtisi* and > 125 in *P. o. wallikeri*. The variant protein isoforms expressed by members of these gene families may be important for interactions with multiple host ligands and, as they are likely to be antigenically variant, their expansion is thought to have been driven by host immune pressure. Expansion of reticulocyte binding-like proteins (RBP), involved in red blood cell invasion, has been observed in both ovale genomes (13–14 genes), gene copy numbers similar to *P. vivax*, while in other species only ~ 2–8 copies have been identified. An expansion of the *Plasmodium* ookinete surface protein P28 appears to be a specific feature of both *P. ovale* spp, as only one copy appears to exist in the genomes of other human-infecting species in the genus.

All the available data confirm that there is a close genetic relationship between the two species, supported by phylogenetic analysis that show *P. o. curtisi* and *P. o. wallikeri* grouping together in the same clade in all studies to date [2, 23, 159]. However, many differences between the two taxa have been observed when comparing *surfin*, *pir* and *rbp* genes, as isoforms with identical sequences have been observed between isolates of the same species, but these families are far more divergent in between-species comparisons of the few *P. o. curtisi* and *P. o. wallikeri* genomes assembled so far. Significant dimorphism has previously been reported in candidate genes across larger datasets from Asian and African isolates [159–161, 175, 185, 210, 211]. For example, specific analysis of nucleotide sequences of five protein-coding regions, likely involved in life cycle sexual stages and so potentially contributing to mating barriers, found that intra-species variation was minimal at each locus, but clear dimorphism were detected when comparing *P. o. curtisi* to *P. o. wallikeri* [181]. Similar results were observed across three vaccine candidate surface proteins in samples collected from Thailand and countries in Africa [185], and in multi-locus sequence analyses reported in a large study of both species in Bangladesh [161]. To better understand the intra- and inter -genetic diversity of these species, more complete reference genomes are needed, as well as a much greater number of isolates undergoing whole genome sequencing across geographic regions.

### Likely origin of these two closely-related, sympatric and non-recombining species

The question as to how two non-recombining sibling species have ended up co-circulating in the same mammalian hosts, transmitted by the same arthropod vectors, has attracted some attention, as has the difficulty in estimating when the two lineages diverged, and in which primate hosts [2, 3, 23, 25, 159]. A thorough summary of the current thinking can be found therein, but the most parsimonious explanation for the current co-circulation of *P. o. curtisi* and *P. o. wallikeri,* in what appears to be perfect sympatry, can be paraphrased from reference 26: pre-ovale parasites in an unknown non-human primate host underwent an initial host transition into hominids some millions of years before the present. This new lineage thus began from a single event, representing an extreme genetic bottleneck, and developed apart from the progenitor stock. Substantial genetic drift occurred, while the two parasite lineages were partitioned in different hosts, a form of allopatry. When a second transition into hominid hosts occurred, again through an extreme genetic bottleneck, both lineages now shared the same hosts, but there was insufficient genetic similarity for fertilisation, meiotic pairing and recombination to occur. However, as the two new species shared almost all features of biology and life history, they together flourished in settings where conditions were favourable and appropriate vectors abundant, and both perished where conditions were harsh. This provides a plausible scenario to explain the contemporary observation that *P. o. curtisi* and *P. o. wallikeri* are now always found co-circulating in the same host and vector populations. Considering these observations, and the irrefutable evidence assembled since 2010 that the ovale parasites represent two distinct sibling species, it is clear that the trinomial nomenclature currently in use is not fit for purpose. Some of the arguments around this can be found in Box 2 of reference 26; to resolve this situation, the current authors and collaborators have developed a proposed solution in which two new binomials are utilized in place of the current nomenclature (manuscript in preparation). In the meantime, correspondence on this topic is most welcome.

As to the evolutionary origins of the ovale parasites, despite twentieth century phylogenetic analyses in general favouring kinship with *P. vivax* [1, 221], genomic sequencing and elucidation of nuclear protein-coding, ribosomal RNA-coding, and mitochondrial genes have more recently placed these species distant from the vivax clade, which includes *P. cynomolgi*, *P. knowlesi* and other SE Asian parasites of simian hosts. Rather a position closer to *P. malariae* [159], Lemuroidea [222], or perhaps the rodent parasite clade [23], have also been put forward. As more genomic information becomes available for *P. o. curtisi* and *P. o. wallikeri* the kinship of these species, and therefore identification of their closest contemporary relatives, should become clearer.

## Concluding remarks

Multi-population genomic studies of the neglected malaria parasites considered here are essential to provide insights into the biology underlying mechanisms of infection, disease progression and adaptation to different hosts. Many questions, for these and other *Plasmodium* species, remain answered, including the ability of some species to form dormant stages in the liver (hypnozoites) as observed for *P. vivax* and *P. ovale* species, and suggested as also possible for *P. malariae* [26], and the regulation of the blood stage cycles that can differ among species (e.g., *P. malariae* has a quartan cycle, a quotidian cycle is observed for *P. knowlesi*, while the other primate species all follow a tertian cycle).

Although genomics studies of these parasites have been difficult, the development of new assays such as SWGA allow the whole genome sequencing of parasite DNA from clinical samples [21], and have therefore opened up new opportunities to understand genomic diversity. Sequencing developments, such as real-time selective sequencing using Nanopore technology, will favour the selection of parasite DNA molecules for sequencing while excluding human molecules [223]. Phenotypic studies of important characters such as drug susceptibility are challenging for these species [224], but the recently developed strategy of “orthologue exchange” now permits detailed in vitro studies of gene function for every species, using transgenic lines with ﻿*P. falciparum* or *P. knowlesi* as the recipient parasite cell. These and future advances can support the large-scale and cost-effective genomic studies of neglected malaria that are now needed. The resulting gains in knowledge will greatly assist the design of species-specific diagnostics, treatments, and surveillance tools, thereby supporting malaria elimination goals.
